# tatDB: a database of Ago1-mediated targets of transfer RNA fragments

**DOI:** 10.1093/nar/gkac1018

**Published:** 2022-11-09

**Authors:** Lingyu Guan, Andrey Grigoriev

**Affiliations:** Department of Biology, Center for Computational and Integrative Biology, Rutgers University, Camden, NJ, USA; Department of Biology, Center for Computational and Integrative Biology, Rutgers University, Camden, NJ, USA

## Abstract

tRNA-derived fragments (tRFs) are a class of emerging post-transcriptional regulators of gene expression likely binding to the transcripts of target genes. However, only a few tRFs targets have been experimentally validated, making it hard to extrapolate the functions or binding mechanisms of tRFs. The paucity of resources supporting the identification of the targets of tRFs creates a bottleneck in the fast-developing field. We have previously analyzed chimeric reads in crosslinked Argonaute1-RNA complexes to help infer the guide-target pairs and binding mechanisms of multiple tRFs based on experimental data in human HEK293 cells. To efficiently disseminate these results to the research community, we designed a web-based database tatDB (**ta**rgets of **t**RFs **D**ata**B**ase) populated with close to 250 000 experimentally determined guide-target pairs with ∼23 000 tRF isoforms. tatDB has a user-friendly interface with flexible query options/filters allowing one to obtain comprehensive information on given tRFs (or targets). Modes of interactions are supported by secondary structures of potential guide-target hybrids and binding motifs, essential for understanding the targeting mechanisms of tRFs. Further, we illustrate the value of the database on an example of hypothesis-building for a tRFs potentially involved in the lifecycle of the SARS-CoV-2 virus. tatDB is freely accessible at https://grigoriev-lab.camden.rutgers.edu/tatdb.

## INTRODUCTION

Transfer RNAs (tRNAs) are best known for their role in translation. There are many databases holding information on tRNAs present in pro- and eukaryotic genomes (1,2), and their identification and curation are not always straightforward (3). In many organisms, the number of tRNA genes is much higher than can be explained by the needs of codon usage (e.g. >500 in the human genome), with some genes being present in many very similar or even identical copies.

Functions of some of the copies of such genes have been recently attributed to generation of tRNA fragments (tRFs), an emerging set of small non-coding RNAs with likely regulatory roles akin to microRNA (miRNA). Just as miRNAs, some of tRFs have been found loaded to Argonaute (Ago) proteins, supporting hypotheses of their potential regulation of RNA targets (4–8). tRFs are very diverse, they can be produced from either the precursor or the mature tRNA, and also from different parts of the latter (most frequently, from the 5′- or 3′-ends, or also from the middle of tRNA). There is additional (smaller) variation in the exact ends of isoforms of otherwise almost identical tRFs, which resembles the case of isomiRs, whereby multiple slightly different miRNAs can arise from a single gene. The Ago loading levels of tRFs differ between cells and also change with age (9), again resembling miRNAs. While numerous fragments are observed in the cells, actual regulatory functions and targets have been experimentally demonstrated only for a few tRFs (5,10–17), and not all of those followed the mode of action of miRNA. Yet given the parallels between tRFs and miRNAs, some experiments validating tRF targeting have been performed with the miRNA ‘seed’ binding in mind (5). However, a large fraction of tRF targets predicted with miRNA tools has failed in validation experiments (10,14), and we have argued that some of the potential binding motifs of tRFs may deviate from the canonical miRNA concepts (6,18).

While the tRF field is relatively new, significant progress has been made to elucidate their types and functions, and several recent reviews offer comprehensive summaries of what is currently known about these molecules (19–25). In addition to human, tRFs have been reported in many other organisms, including mouse (26,27), rat (28), fruit fly (9,29), plants (30,31) and prokaryotes (4). In human cells, tRFs have been found to play a role in cancer-related processes, regulating ribosome biogenesis (17) or displacing RNA-binding proteins (32). In other disease contexts, virus infection has been reported to induce some host tRFs (13,16,33) suggesting a potential implication of tRFs in the host-virus interaction.

An increasing interest in tRFs has led to generating long lists of them, detected in various small RNA sequencing experiments. Several databases hold such lists. However, these databases currently neither offer information on the mechanism of action of these fragments nor on their targets. Experimental evidence of modulating expression of targets and data on a potential binding region exists for only a few tRFs. Most experimental studies focus on one-two tRFs and such results are very hard to generalize (5,10–12,14–16). In contrast, large numbers of tRFs have been detected in small RNA sequencing datasets and simply listed in the databases. For example, there are >28 000 of human tRFs in MINTbase v2.0 (34), many of which often differ by only one nucleotide. This discrepancy between excessive tRF numbers and the lack of understanding of their function hinders hypothesis generation and prevents efficient systematic functional testing of these novel regulatory molecules. Recently, several databases addressing such issues have appeared (35–38). While attempting to connect tRFs to their targets, these databases contain only relatively small subsets, omitting large numbers of interactions detected (and also, importantly, those validated) experimentally, and we provide more detailed information in Discussion.

To bridge such gaps, we developed a database of targets of tRFs (tatDB). We aimed to create a transcriptome-scale resource and selected for this purpose a dataset, initially produced as a set of chimeric reads combining small RNA guides and mRNA targets, with a focus on miRNAs (39). This dataset/method is called CLASH (40) and our analysis of it is further described in the Methods. Importantly, CLASH contains information about other small RNAs paired with mRNA targets (4). We comprehensively re-analyzed this dataset and observed >8 million reads supporting the interactions between Ago1-loaded tRFs and various types of target RNAs, which is a significant extension to the other studies. We identified >1000 tRFs (∼23 000 isoforms), including ones validated by independent experiments (5,10–16). We used these tRF–target CLASH pairs to infer binding regions (motifs) in many tRFs (6), and our results matched the validated binding regions for Ago-mediated targeting in well-studied tRFs (5,12). Such binding regions in tRFs were also spatially compatible with the Ago-crosslinking sites in tRFs that we identified in PAR-CLIP datasets (41) and included in tatDB. With comprehensive and stringent analyses, we incorporated many tRFs missing from the existing databases (35–38). Some of these have been experimentally validated, e.g. a tRF derived from the IleTAT 3′ trailer (12). We also detected and included in tatDB novel intron-derived putative tRFs that invite further investigation.

It is important to note the limitations of the presented data. CLASH is a single dataset, albeit a large one. In our detailed analyses of CLASH (6,18) we have also reported previously unknown biases/features in the reads produced by this technique. While the models we have proposed (6,18) may account for some biases there is no guarantee that no other biases have been missed. The experimental readouts in CLASH (and also PAR-CLIP) may be affected by protein expression artifacts and the results may not reflect tRF loading (and detectable pairing with targets) corresponding to the endogenous levels of Ago1 *in vivo*. Further, post-lysis loading to Ago of miRNAs (42) (and possibly other small RNAs) may occur and is seldom discussed in the papers on large-scale RNA-protein interactions, although lowered by the cross-linking approaches (43). To summarize, analysis of such data should take into account the numerous sources of potential error.

We reasoned that tatDB would be a convenient tool for browsing and searching CLASH results (otherwise available as millions of reads requiring complex processing) for potential targets and binding regions as well as for evaluating whether certain hybrid pairs represent biological signal or noise. We hope it would help drive further experiments to establish the actual function and targets of tRFs. With this motivation, we have made it into a publicly available database.

## MATERIALS AND METHODS

### Building tRNA index

We downloaded tRNA genes from GtRNAdb (1), tRNAdb and mitoRNAdb (2). We firstly ran Bowtie 1.0.1 (-v 0 -a) to align all tRNA genes to human genome (hg38) and excluded sequences that could not be mapped to nuclear or mitochondrial genome. Remaining tRNA genes were collapsed and reindexed for every isodecoder.

In order to identify tRFs from pre-tRNAs, we extended 40-nt genomic sequences downstream of the tRNA genes. tRNA isoforms that could be mapped to multiple genomic locations may have different flanking sequences on the genome and we considered all possible 3′ trailers.

tRNAs with introns in the genes were considered in mature (intron was removed from the sequence) or immature (intron was retained the sequence) state for the origins of tRFs. tRFs that cross the exon-exon junctions have ‘X’ in their IDs (see an example in Results section) to indicate the introns were crossed out from the sequences.

### Analyses of CLASH dataset and PAR-CLIP dataset

Cross-Ligation and Sequencing of Hybrids (CLASH) method has been developed to identify the chimeric sequences corresponding to RNA-RNA duplexes hybridized *in vivo* and crosslinked with Ago1 proteins in the human HEK293 cells (39,40). We performed stringent and comprehensive analysis of the CLASH data to establish Ago1-associated tRFs-targets pairs from these chimeric sequences, to identify noise and bias in these datasets, as described previously (6,18), and to extract potential interactions of higher confidence between tRFs and targets.

The short sequences of tRFs present multiple challenges for sorting out and presenting the CLASH data: e.g. how to handle guides exactly mapped to multiple tRNA genes or how to deal with exon-intron borders? Examples of noise or bias included random sequences matching parts of tRNA and/or target and co-occurring close each other in the genome or a transcript, or polyT tracts matched to certain mRNAs in some chimeras, etc, as described earlier (18).

Using different targets of the same tRF, we identified common statistically significant motifs, which likely correspond to tRF–target binding regions (at least for several known cases (5,12)) and present a strong argument against their random pairing in CLASH (6). In tatDB, we report various estimates of confidence in interactions, e.g. based on finding the same interaction pairs in divergent and unique chimeras/hybrids. Further, many tRFs were found on both ends of the chimeric reads with target sequences on the other respective end. We call chimeras ‘Forward’ for tRFs on the 5′ end and ‘Reverse’ for tRFs on the 3′ end. Chimeras with such divergent orientations are independent biological constructs and they often support the same tRF–target pairs and common binding motifs in the tRFs (18).

Given the small size of the mitochondrial genome, we observed a few cases when the target sequence was close to a tRNA gene. For completeness, we kept all such mitochondrial pairs and polyT-containing hybrids. However, some of them (tRFs resulting from the tRNA 5′ end and trailer sequence are found in CLASH pairs with adjacent targets) may represent CLASH artifacts, hence we provided a warning in the Help section of the tatDB.

Potential Ago-crosslinking sties were revealed by T > C conversions in PAR-CLIP datasets of human Ago1–Ago4 (41). PAR-CLIP identified conversions were mapped to tRFs identified by CLASH using our earlier approach (18).

### Naming convention for tRFs

We devised a tRF naming scheme that is informative and extensible It has this format:

***AA Anticodon*** ***Gene*** ***Genome Isoform Type(X) Start End***

For example,

***LeuAAG-001-N-3p-68* *–* *85***

Every tRNA isoform was assigned with a unique ID using Amino Acid (AA)_Anticodon followed by a three-digit index of the corresponding tRNA gene, and N or M indicating such gene was encoded on nuclear genome or mitochondrial genome. tRFs which could be mapped to tRNAs in both nuclear and mitochondrial genome had -NM- in their IDs.

We separated tRFs into six types based on their cleaved positions on reference sequences. tRFs which have their 5′ borders cleaved in the first five nucleotides of tRNAs were classified as tRF-5. If the 3′ border of a tRF-5 was in the anticodon loop of a tRNA molecule, it was considered as a 5′ tRNA half and was hence named as tRF-5i in our database. tRF-5 with its 3′ border being cut upstream of the anticodon loop was called tRF-5p. Similarly, tRFs with their 3′ borders cleaved in the last five nucleotides of mature tRNAs (including CCA addition) were classified as tRF-3. tRF-3 were further separated to tRF-3i (3′ tRNA half) or tRF-3p. tRF with its 3′ end being cleaved in the 3′ trailer sequence of a pre-tRNA was defined as tRF-3t. The 5′ border of a tRF-3t could be either within the tRNA gene or in the trailer. tRFs were called tRF-Mi if their 5′ and 3′ ends were in the internal regions (excluding first and last 5 nts) of tRNAs.

tRNAs isoforms transcribed from the same gene with or without intron retention have the same three-digit index. The coordinates of tRFs on the tRNA isoforms were always calculated based on the sequence of tRNA genes (introns were retained). We added a letter ‘X’ after the tRF type (e.g. IleTAT-001-N-iX-36–76) to indicate the tRFs were derived from mature tRNAs where the introns were removed, and to discriminate such tRFs from the tRFs have the same coordinates but the introns were retained in the sequence (e.g. IleTAT-001-N-i-36–76).

Some tRFs may be aligned to various tRNA isoforms and even different tRNA isoacceptors. Although these tRFs could have different IDs, we provided only one representative ID based on the tRNA isoform which has most copies on the genome unless specifically indicated on the result page.

## RESULTS AND DISCUSSION

We implemented tatDB as MySQL database with a user-friendly interface, allowing for flexible queries using multiple parameters both for tRF and target sequences. The database is publicly available at https://grigoriev-lab.camden.rutgers.edu/tatdb.

### Search and filters

**Figure 1.**
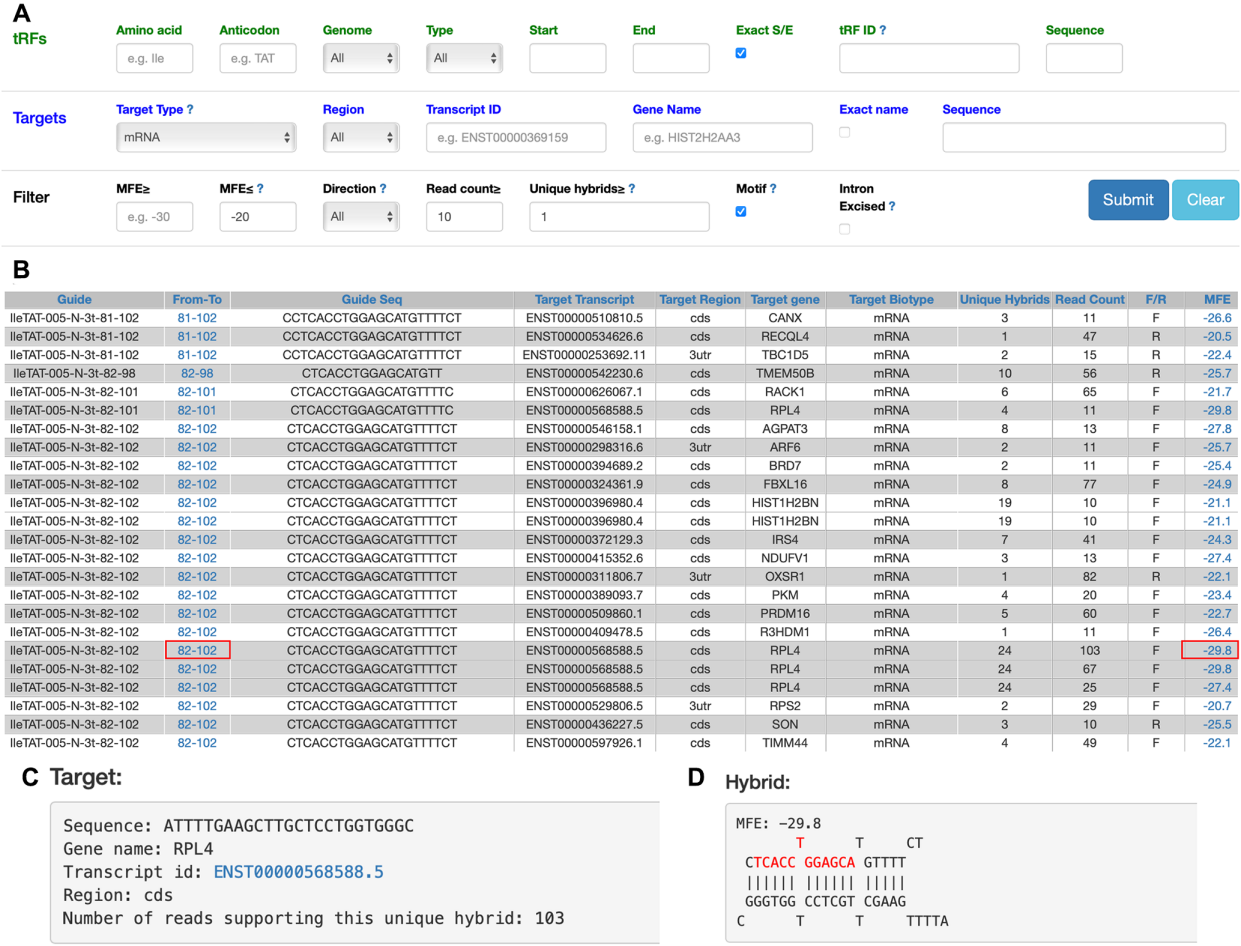
Search functionality in tatDB. (**A**) The main search page with default filters indicated. (**B**) Output of the search results for IleTAT tRF-3t. MFE value in red box hyperlinks to (C) and (D). Coordinate in green box hyperlinks to Figure 2. Download link (not visible here) is available at the bottom of the page. (**C**) Details of the unique hybrid. (**D**) Secondary structure/hybridization pattern of the tRF–target pair. F/R: forward/reverse hybrid orientation.

The query front end of tatDB is shown in Figure 1A. We provide multiple options to apply different filters to (i) query tRFs to find their targets or (ii) query targets and find tRFs. At the top level, amino acid (3-letter standard abbreviation, AA) and anticodon can specify the tRNA host genes for tRFs. ‘Genome’ indicates whether the tRNA gene is encoded in the nucleus (N) or mitochondria (M), or if a tRF can be attributed to both (NM). If ‘Exact S/E’ is checked, tRFs with exact start and end positions on the reference tRNAs are returned. Otherwise, all tRFs that are included in the range [Start:End] will be shown. Partial input of ‘tRF ID’ (as defined in Materials and Method) is allowed. tatDB also allows users to search tRFs by sequence, tRFs that contain the entire input sequence without mismatches will be displayed.

Various filters on targets could be specified to query target RNAs crosslinked with tRFs *in vivo* in Ago1. Many RNA types have been found paired with tRFs in Ago, and although the biological interpretation of pairs with non-mRNAs is unclear, we included them for completeness. Thus, different target types including mRNA, rRNA, miRNA and lincRNA may be selected. Other types of transcripts annotated in Ensembl genome browser 91, such as snoRNA, snRNA, pseudogene (PG) and processed transcript (PT), are grouped as ‘Other’. The Region filter allows one to find targets in exons (including 5′ UTR, CDS and 3′ UTR) or intronic regions. Users can look for interactions of tRFs with a given gene; if ‘Exact name’ is unchecked, partial input of ‘Gene Name’ is allowed. Targets can be queried using a Sequence field, target RNAs containing the complete input sequence are returned with their paired tRFs.

Additional query criteria include: (i) range of the minimum free energy (MFE) of the interaction of tRF and target sequence, (ii) direction of the pair of tRF and target RNA in the CLASH chimeric read as defined earlier (18), (iii) minimum number of CLASH reads supporting every unique hybrid, (iv) minimum number of unique hybrids for a target gene, (v) tRFs containing motifs. Based on the tRF parameter distribution and to show high-confidence interactions, we provided some useful default filters (target type = mRNA, MFE ≤ −20, read counts ≥ 10 and motif = true), which can be removed by clicking on the ‘Clear’ button. Setting more permissive thresholds here could be useful for finding tRF–target pairs of lower confidence, if needed.

### List of tRF–target pairs

Search results are output in a text table, where every line represents a unique hybrid found in CLASH between a tRF sequence and a target sequence (Figure 1B). In many cases, CLASH chimeras for a given tRF have been found to contain overlapping subsequences of the same target gene, different by only a few end nucleotides. Such pairs of a tRF and different subsequences of the same gene are considered as different unique hybrids and presented in lines with the same background color in the result table. Additionally, counts of forward and reverse chimeras for the same pair are listed. The MFE value is a hyperlink to a page with the secondary structure of the given tRF–target pair and other details of the unique hybrid (Figure 1C, D).

### List of all target genes for a given tRF isoform

**Figure 2.**
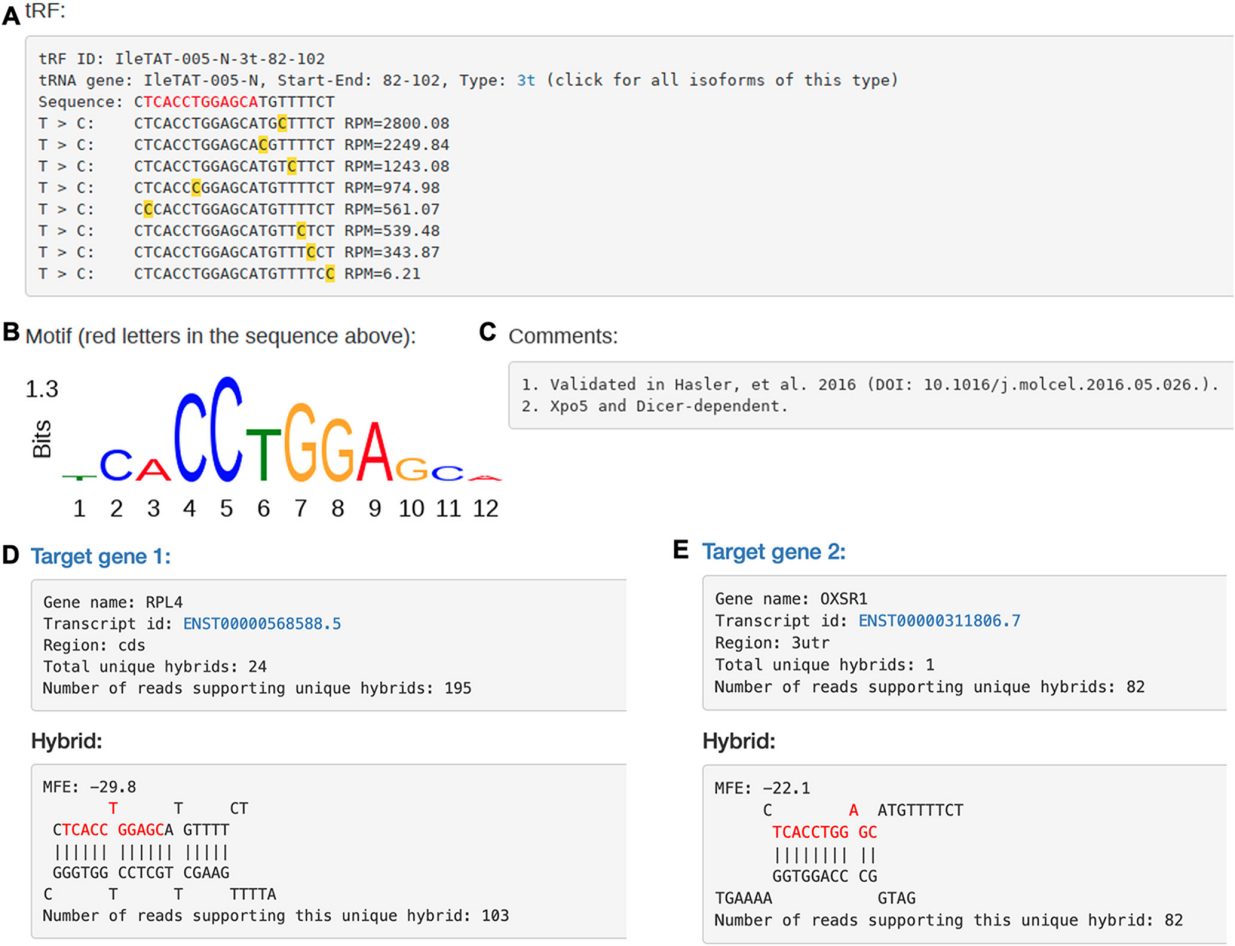
Detailed information of tRF IleTAT-005-N-3t-82–102. (**A**) tRF sequence, origin and T > C conversions identified in PAR-CLIP datasets. (**B**) Motif logo. (**C**) Manual curation in free text, currently accessible to administrators, may be submitted for review/use. (**D**, **E**) Top 2 target genes for this tRF, with links to Ensembl and likely hybridization patterns.

A column ‘From-To’ refers to the start and end of a tRF: for a given tRNA gene this coordinate uniquely identifies a tRF. A hyperlink in this column directs users to a page with detailed information for such tRF, including its sequence, host gene and PAR-CLIP matches (Figure 2). If a motif was identified for this tRF (6,18), its logo is shown and the motif is highlighted in red in the tRF sequence. T > C conversions (sites of likely Ago cross-linking, usually not involved in the target binding) identified in human PAR-CLIP datasets are underlined next to their frequencies (Figure 2A). Due to the discrepancies in the library preparation steps of different experiments which cause tRF length variation, we aligned T > C conversion sites in PAR-CLIP datasets to tRFs identified in CLASH (18), since they seem to appear in the same locations in different isoforms. Thus, although T > C conversions are shown for a particular tRF isoform, it is to simply indicate their positions relative to the motif, as that exact tRF isoform is not always observed in PAR-CLIP datasets.

**Figure 3.**
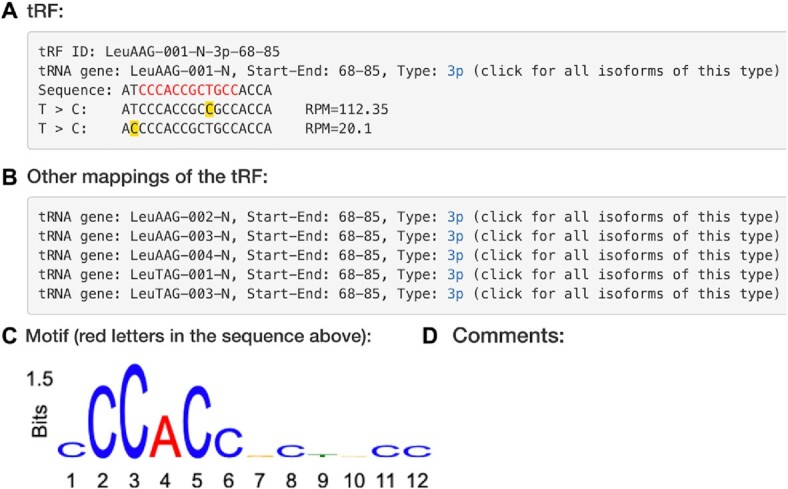
Detailed information of tRF LeuAAG-001-N-3p-68–85. (**A**) tRF sequence, origin and T > C conversions identified in PAR-CLIP. (**B**) Mappings of the tRF to other tRNA isoforms. (**C**) A logo for the motif identified using CLASH identified pairs of tRFs and targets. (**D**) Space reserved for manually curated comments, see Figure 2.

If a tRF isoform could be mapped to different tRNAs, a section of all alternative mappings will be shown (Figure 3). As an example, we take one of the most studied tRF LeuAAG-001-N-3p-68–85 (tRFdb ID: 3001a), which can also be mapped to the 3′ end of LeuTAG tRNA. Hyperlinks to the different tRNAs are provided to show the mapping of such tRF to the tRNA along with other tRFs-3p (see more details in next Section).

Target genes binding to this tRF isoform are listed below (Figure 2D, E). Filters selected in the search page are automatically applied and they may be modified on this page to show specific set of target genes, e.g. only mRNA targets. In many cases, a gene may be targeted by a tRF in multiple positions. On this page, only one target position which has strongest pairing with the tRF (with minimum MFE) is shown. Gene name hyperlinks to a new page to view all target sites and their hybrids with the tRF.

### Database interoperability, linking to tatDB

As different sources of data on tRFs appear, it is important to allow for collecting facts about these molecules across databases. Currently, different databases of tRFs have their own naming conventions, e.g. 3001a in tRFdb is named tRF-18-HR0VX6D2 in MINTbase. Such tRF IDs are hard to interpret or match and they may cause difficulties in linking them. To allow for meaningful connections, we provided a simple and reliable mechanism based on a common currency of tRFs—their sequence. If the sequence of a tRF is known, one can simply link from any other source to its tatDB page with details of the tRF and all target genes by referring to the page (such as shown in Figures 2 and 3) using the tRF sequence in the URL: https://grigoriev-lab.camden.rutgers.edu/tatdb/trf_isoform.php?guide_seq=ATCCCACCGCTGCCACCA.

To view all binding sites on a gene targeted by a known tRF, one can refer to the page with the tRF sequence and gene name in the URL, for example: https://grigoriev-lab.camden.rutgers.edu/tatdb/trf_gene.php?guide_seq=CTCACCTGGAGCATGTTTTCT&gene_name=RPL4.

Default filters will be selected for such links if no filter is specified in the URL or in the previous search step. Further, filter settings may be changed after landing on the tatDB page.

### Alignments of all identified tRFs to a given tRNA sequence

**Figure 4.**
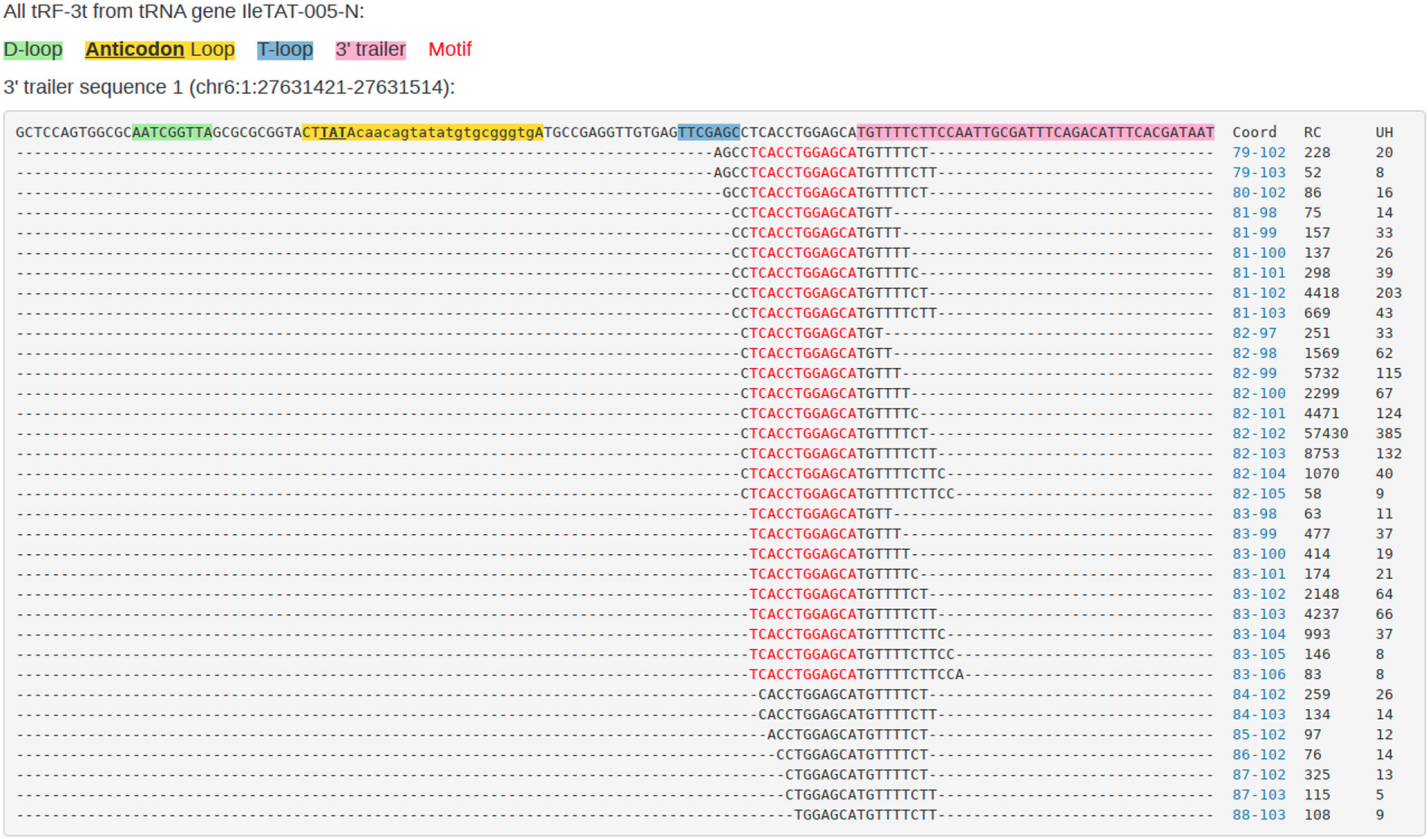
Alignments of tRF-3t supported by >50 reads to the 3′ trailer sequence of IleTAT. Red letters in the sequence indicate putative binding motifs. RC: read count, UH: unique hybrids.

Hyperlink of the tRF type in Figure 3 directs to a page to show the alignments of a specific type of tRFs to a given tRNA gene (Figure 4, Supplementary Figure S1 & Supplementary Figure S2). In-page filters can be set to view tRF isoforms with certain read count and unique hybrids. tRF-3t to different 3′ trailers of a given tRNA isoform are shown separately on the same page (Supplementary Figure S1).

In order to identify all possible tRFs from either mature or immature tRNAs, we include tRNAs sequences with or without introns in the reference database to search tRFs (see Materials and Method). We herein showed an example that tRF-i could origin from both exon–intron junctions and exon–exon junctions in IleTAT-005-N (Supplementary Figure S2) although the significance of such tRFs remain to be elucidated further by experiments.

## DISCUSSION

Some recently described resources have attempted to catalogue tRF and target interactions. In tRFtar (36) and tRFtarget (38), tRFs have been retrieved from existing databases for tRFs (34,44) with binding targets computationally predicted in the whole human transcriptome in tRFtarget. It appears that only five tRF–target interactions observed in CLASH have been manually curated in tRFtarget (38). Targets in tRFtar (36) have been further restricted to those found in CLIP datasets. Although 772 tRF–target interactions in CLASH have been included (yet not available for querying), none of the five most abundant interactions curated in tRFtarget (38) could be found in tRFtar (36) at the time of writing this paper.

Also, these two databases rely on the tRFs annotated in existing databases, and that presents a major limitation. For example, MINTbase (34) seems to contain only tRFs derived from mature tRNAs. A limited number of tRFs derived from the 3′ trailer sequence of pre-tRNAs are annotated in tRFdb (44) but at least one of the experimentally validated tRF-3t (12) is not in it. The other two recently published databases, tsRBase (35) and tsRFun (37), have identified tRFs *de novo* by aligning reads in small RNA sequencing datasets to tRNAs from GtRNAdb (1). tsRBase (35) identified 3,298 tRF–target interactions in CLASH and CLEAR but neither the five curated CLASH interactions in tRFtarget (38) nor the abundant tRF-3t (12,18) have been detected in this study. The same problem exists for tsRFun (37) in that the tRF–target interaction set they identified from CLASH data was far from complete. Another limitation is that none of the databases above specifically focuses on the tRF binding motifs, which could provide insights into the mechanism of interaction with tRF targets and explain the biology of these novel potential regulators.

Validated tRF–target interactions are currently limited compared to the large number of published CLIP-seq or RNA-seq datasets, which can detect only guide or target RNA molecules but not their pairs. Thus, a computational prediction of tRF targets is a widely employed strategy to investigate the functions of tRFs. Based on the assumption that tRFs act akin to miRNAs, several miRNA target predictors have been used for tRFs, e.g, TargetScan in Maute *et al.* (10), miRanda in Zhang *et al.* (14) and RNA22 in Kuscu *et al.* (5), all with a high proportion of false positives. RNAhybrid has been utilized to calculate the MFE for tRFs binding to target candidates, selected, e.g. from transcripts co-expressed with the tRFs, or from the whole mRNA transcriptome (35–38), also with limited success in predicting interactors.

Without a high-confidence true positive dataset for algorithm training, these unsupervised methods apparently have high false positive rates resulting in most predictions failing to be experimentally validated. tatDB provides a variety of features for the tRF–target pairs detected in the Ago1 and flexible query interface for the users to quickly pick up high-ranked tRFs for experiments. Moreover, these features and scores (e.g. the sequences, binding sites and their corresponding motifs) for numerous tRF–target pairs in tatDB may serve as a basis of high-value true positive set for developing a tRF-specific target predicting algorithm, urgently needed in this field of study.

Different tRNA isodecoders and isoacceptors have common sub-sequences of high similarity. To identify all possible tRFs, those tRNA sequences are all included in the search database. While others have often employed similar strategy of having a redundant search database, the advantage of tatDB is that we clearly list all mappings (Figure 3B) for a given tRF and their coordinates in each tRNA isoform can be easily traced and viewed on the graphical alignment page (Figure 4). In addition, tRNA genes are highly repeated on the genome. Most identical tRNA isoforms also have identical 3′ trailer sequences, yet there are some exceptions (e.g. HisGTG tRF-3t as shown in Supplementary Figure S1), where the trailer sequences are different for tRNA genes with identical body sequences. Our database provides a user-friendly means to view the origins of such tRFs graphically.

As tRF-3t have been reported to be generated from the 3′ trailer sequences of pre-tRNAs, one should consider the pre-tRNAs as the source of other types of tRFs. In most existing databases (35–38), tRNA introns are simply removed from the tRNAs and only mature tRNA sequences are used as reference to look for tRFs. We observed tRFs mapped to tRNA introns (18), although their significance is yet unclear due to the paucity of sequenced reads (and hence lack of motifs) for such cases in the available CLASH data. Our database is the first attempt to clearly represent all those intron-derived tRFs so that they can be easily compared to the tRFs spliced cross the exon-exon junctions (Supplementary Figure S2).

As an additional illustration of the utility of tatDB, we provide here an example of a possible hypothesis generation for interpreting of the results of the current active research in coronavirus. We have noted earlier various factors linking the features of the SARS-CoV-2 genome with short RNAs (45), and were also intrigued by an observation of a significant increase in tRF levels in patient samples (46). Although the tRF IDs in that paper do not match ours, we performed a sequence search of a highly upregulated (∼35 fold) tRF5-Leu-AAG in tatDB and identified RPL18A as a potential target. RPL18A showed the highest number of unique hybrids (for sequence GGUAGCGUGGCCGAGC and two other isoforms extended by 1 and 2 nts), supported by the highest number of reads, compared to other targets. Following up on the target, intriguingly, we found that this protein may play a role in translation of another RNA virus (hepatitis C virus) by interacting with its internal ribosome entry site (47). Given the reorganization of host translation by both viruses, could this link ‘tRF > RPL18A > translation’ be relevant for the SARS-CoV-2 life cycle as well? Although the final answer should be produced by an experiment, this hypothesis is generated from a straightforward use of tatDB, and we expect multiple similar cases of utilization of this resource by the research community.

## DATA AVAILABILITY

All results of this study are freely accessible on the database website, https://grigoriev-lab.camden.rutgers.edu/tatdb/.

## Supplementary Material

gkac1018_Supplemental_File
