# Supplementary material for: tatDB: a database of Ago1-mediated targets of transfer RNA fragments
Source: Nucleic Acids Res. 2022 Nov 9;51(D1):D297–305. doi: 10.1093/nar/gkac1018 (PMC9825446; doi:10.1093/nar/gkac1018)
Supplement: gkac1018_Supplemental_File [file gkac1018_supplemental_file.pdf]

Supplementary Figures

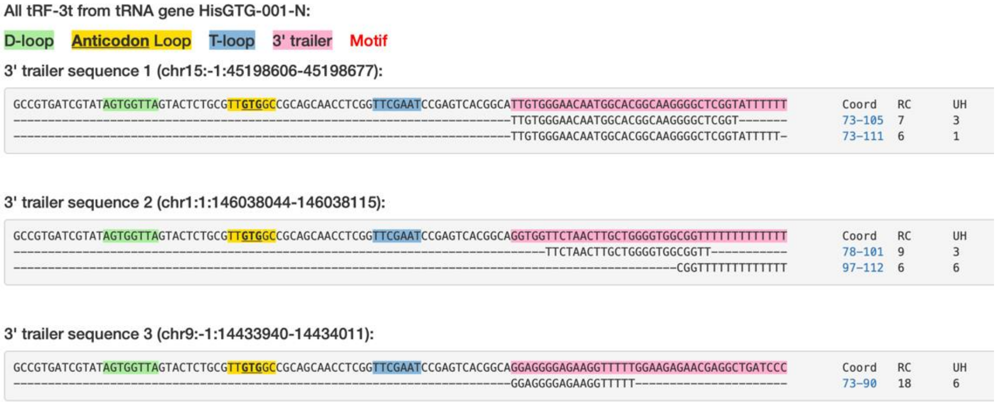

Supplementary Figure 1. Alignments of tRF-3t supported by >5 reads to the HisGTG. The tRNA gene has multiple gene copies on the genome. All possible 3' trailer sequences from different loci are shown for tRF alignments.

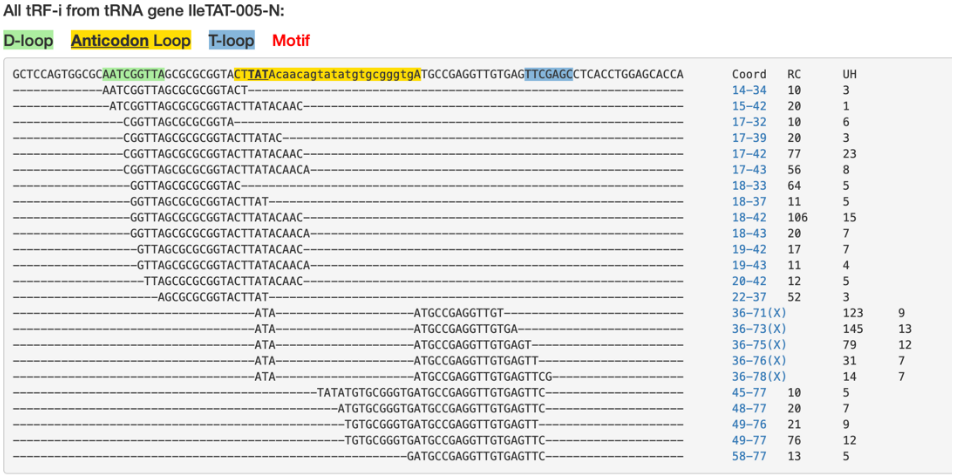

Supplementary Figure 2. Alignments of tRF-i supported by >10 reads to the IleTAT. Intron retention in the tRNA sequence is shown in lower case. The coordinates of tRFs are calculated based on the sequence of tRNA genes. tRFs with "X" in the coordinate column and IDs derive from mature tRNAs with introns removed from the sequence.
